# EUKARYOME: the rRNA gene reference database for identification of all eukaryotes

**DOI:** 10.1093/database/baae043

**Published:** 2024-06-12

**Authors:** Leho Tedersoo, Mahdieh S Hosseyni Moghaddam, Vladimir Mikryukov, Ali Hakimzadeh, Mohammad Bahram, R Henrik Nilsson, Iryna Yatsiuk, Stefan Geisen, Arne Schwelm, Kasia Piwosz, Marko Prous, Sirje Sildever, Dominika Chmolowska, Sonja Rueckert, Pavel Skaloud, Peeter Laas, Marco Tines, Jae-Ho Jung, Ji Hye Choi, Saad Alkahtani, Sten Anslan

**Affiliations:** Mycology and Microbiology Center, University of Tartu, Liivi 2, Tartu 50400, Estonia; Institute of Ecology and Earth Sciences, University of Tartu, Liivi 2, Tartu 50400, Estonia; Department of Zoology, College of Science, King Saud University, PO Box 2455, Riyadh 11451, Saudi Arabia; Institute of Ecology and Earth Sciences, University of Tartu, Liivi 2, Tartu 50400, Estonia; Mycology and Microbiology Center, University of Tartu, Liivi 2, Tartu 50400, Estonia; Institute of Ecology and Earth Sciences, University of Tartu, Liivi 2, Tartu 50400, Estonia; Institute of Ecology and Earth Sciences, University of Tartu, Liivi 2, Tartu 50400, Estonia; Institute of Ecology and Earth Sciences, University of Tartu, Liivi 2, Tartu 50400, Estonia; Department of Ecology, Swedish University of Agricultural Sciences, Ulls väg 16, Uppsala 75651, Sweden; Department of Biological and Environmental Sciences, Gothenburg Global Biodiversity Centre, University of Gothenburg, Box 461, Göteborg 40530, Sweden; Institute of Ecology and Earth Sciences, University of Tartu, Liivi 2, Tartu 50400, Estonia; Laboratory of Nematology, Wageningen University, Droevendaalsesteeg 1, Wageningen 6708PB, The Netherlands; Laboratory of Nematology, Wageningen University, Droevendaalsesteeg 1, Wageningen 6708PB, The Netherlands; Department of Environment, Soils and Land-Use, Teagasc, Oak Park House, Wexford R93 XE12, Ireland; National Marine Fisheries Research Institute, Kołłątaja 1, Gdynia 81332, Poland; Institute of Ecology and Earth Sciences, University of Tartu, Liivi 2, Tartu 50400, Estonia; Ecology and Genetics Research Unit, University of Oulu, Box 8000, Oulu 90014, Finland; Department of Marine Systems, Tallinn University of Technology, Mäealuse 14a, Tallinn 12618, Estonia; Institute of Systematics and Evolution of Animals, Polish Academy of Sciences, Sławkowska 17, Kraków 31016, Poland; Eukaryotic Microbiology, Faculty of Biology, University of Duisburg-Essen, Universitätsstraße 1, Essen, Nordrhein-Westfalen 45141, Germany; Department of Botany, Faculty of Science, Charles University, Benatska 2, Praha 12800, Czech Republic; Department of Marine Systems, Tallinn University of Technology, Mäealuse 14a, Tallinn 12618, Estonia; Institute of Technology, University of Tartu, Nooruse 1, Tartu 50400, Estonia; Department for Biological Sciences, Institute for Ecology, Evolution, and Diversity, Goethe University Frankfurt am Main, Max-von-Laue-Str. 13, Frankfurt am Main 60438, Germany; Senckenberg Biodiversity and Climate Research Centre, Georg-Voigt-Straße 14-16, Frankfurt am Main 60325, Germany; Department of Biology, Gangneung-Wonju National University, Jukheon-gil 7, Gangneung 25457, South Korea; Department of Biology, Gangneung-Wonju National University, Jukheon-gil 7, Gangneung 25457, South Korea; Department of Zoology, College of Science, King Saud University, PO Box 2455, Riyadh 11451, Saudi Arabia; Institute of Ecology and Earth Sciences, University of Tartu, Liivi 2, Tartu 50400, Estonia

## Abstract

Molecular identification of micro- and macroorganisms based on nuclear markers has revolutionized our understanding of their taxonomy, phylogeny and ecology. Today, research on the diversity of eukaryotes in global ecosystems heavily relies on nuclear ribosomal RNA (rRNA) markers. Here, we present the research community-curated reference database EUKARYOME for nuclear ribosomal 18S rRNA, internal transcribed spacer (ITS) and 28S rRNA markers for all eukaryotes, including metazoans (animals), protists, fungi and plants. It is particularly useful for the identification of arbuscular mycorrhizal fungi as it bridges the four commonly used molecular markers—ITS1, ITS2, 18S V4–V5 and 28S D1–D2 subregions. The key benefits of this database over other annotated reference sequence databases are that it is not restricted to certain taxonomic groups and it includes all rRNA markers. EUKARYOME also offers a number of reference long-read sequences that are derived from (meta)genomic and (meta)barcoding—a unique feature that can be used for taxonomic identification and chimera control of third-generation, long-read, high-throughput sequencing data. Taxonomic assignments of rRNA genes in the database are verified based on phylogenetic approaches. The reference datasets are available in multiple formats from the project homepage, http://www.eukaryome.org.

## Introduction

Accurate taxonomic identification of organisms is one of the cornerstones of biology and its many subdisciplines, including ecology, biogeography, phylogenetics and conservation biology. Since the eighteenth century, descriptions of new species and comparisons among species have been based on macroscopic and microscopic characters. Over two centuries of taxonomic work have set a firm basis for our alpha taxonomic (species-level) knowledge (1). However, distinguishing among species of microorganisms (including microfauna) using only morphological features has proved more challenging (2). Since the development of Sanger sequencing in the late 1980s, ribosomal RNA (rRNA) genes and the intercalary internal transcribed spacer (ITS) region have served as the main molecular markers for providing additional non-morphological characters for species discrimination. They are also routinely used for phylogenetic studies and identification of unknown organisms from propagules lacking taxonomically informative characters, pure cultures of microorganisms and environmental DNA (eDNA) (3).

While the identification of prokaryotes has almost exclusively used the small subunit rRNA (16S rRNA, SSU) gene, the situation is much more varied for eukaryotes, where the choice of markers often depends on the target groups. For example, the cytochrome c oxidase subunit 1 gene is the marker of choice for metazoans (animals *s.lato*), and different plastid markers are used in plants and algae. However, for most other groups of eukaryotes including protists and fungi, eDNA-based research relies on rRNA genes and the ITS region, thereby benefiting from high-coverage primers, high copy number in genomes and the availability of ample reference data (4). Traditionally, many metazoan groups such as nematodes, many protist groups and unicellular fungal phyla have primarily been assessed using the most conservative rRNA gene, namely the SSU. In contrast, the large subunit rRNA (28S rRNA, LSU) gene is widely used in the study of certain invertebrate groups, algae, alveolates, chytrids and arbuscular mycorrhizal fungi. The ITS region—the least conserved of these markers—is commonly used for ascomycetes and basidiomycetes, plants, oomycetes, ciliates, dinoflagellates and certain hexapod groups. A few exceptions aside, these rRNA marker regions can be amplified and sequenced for all groups of eukaryotes, but attempts at species- or genus-level identification may suffer from poor resolution or paucity of reference data for the particular marker fragment.

Extant and emerging long-read high-throughput sequencing methods generate much more sequence data and thus taxonomic precision compared to short-read high-throughput sequencing methods (5–7) but require high-quality, long-read reference data to maximize their potential. While the International Nucleotide Sequence Databases consortium (INSDc) contains much of the Sanger sequencing data and genomic data, the entries are commonly misidentified (as a result of contamination or taxonomic mislabeling) or named as uncultured organisms, with limited options for third-party re-annotation (8). Tailored and taxonomically curated reference databases typically focus on one single ribosomal marker—either SSU, LSU or ITS—and tend to be of a narrow taxonomic scope. For example, the UNITE database covers all eukaryotes, but its functionalities are restricted to the ITS region (9, 10). The PR2 database (11) is focused on the SSU gene of mostly aquatic protists. The SILVA database (12) includes both SSU and LSU genes, but its most recent version dates back to 2020 (https://www.arb-silva.de/). Both SILVA and PR2 include data from amplicons and (meta)genomes. It is noteworthy that none of the reference databases use long reads spanning the SSU, ITS and LSU regions, which would be essential for chimera filtering and improved identification of ultra-long reads (13).

Here, we introduce a curated eukaryote-wide reference database—EUKARYOME—which compiles well-annotated, non-redundant, high-quality reads for the SSU, ITS and LSU marker subsets—separately and combined—for high-accuracy taxonomic reference and chimera recognition. EUKARYOME includes rRNA genes from recent (meta)genomics and (meta)transcriptomics and long-read (meta)barcoding studies. The original taxonomic annotations are updated based on phylogenetic approaches and information in recently published databases and datasets, as well as changes in phylogeny-based hierarchical taxonomy. The reference datasets are freely available for download from the EUKARYOME homepage (http://www.eukaryome.org) in several widely used formats to facilitate their implementation in various bioinformatics pipelines and platforms for sequence-based identification, chimera filtering and phylogenetic analyses.

## Materials and methods

Initially, we compiled nuclear rRNA SSU, ITS and LSU sequence data from SILVA v138.1, PR2 v4.14.1 and UNITE v9.0 databases along with their taxonomic annotations. Since the reads were typically trimmed to remove the flanking marker(s), we downloaded the full-length reads from the INSDc on 16 February 2023. We also downloaded all reads annotated to the level of species that were published since 2018 to reflect the most recent, high-quality taxonomic data. In addition, we included long-read amplicons from PacBio-sequenced samples of various environments (13; M. Hosseyni Moghaddam *et al*., unpublished results), soil (14; M. Sharma *et al*., unpublished results), marine water (15) and animal rumen (16). From each of these studies, we used representative sequences of operational taxonomic units as provided by the authors. Although these individual studies performed comprehensive quality-filtering, we subjected these PacBio sequences to an extra round of chimera control using the software ITSx v1.1.2 (17) and UCHIME v4.2 (18) with default options and UNITE, PR2 and SILVA as references. We also performed additional quality-filtering based on the distribution of indels: first, reads with up to two gap (indel) openings relative to the closest sequence were retained; then, in the remaining sequences, only those were retained where the number of substitutions exceeded the number of indels. For the retained reads, we performed multiple sequence alignments in batches of up to 5000 reads using MAFFT v7 with standard options (19). The alignments were visualized in AliView v1.26 (20). Sequences with at least two indels in one of the five highly conserved regions over a window of 240 bases were flagged as low quality and removed. As a result of these procedures, 15–25% of PacBio reference sequences were excluded depending on the dataset. Although PacBio HiFi reads are on average >99.9% accurate, a small but significant proportion of reads can be expected to suffer from artificial indels in spite of their relatively high-quality scores, hence our unforgiving scrutiny of the PacBio sequences. Oxford nanopore consensus reads (at least 20× coverage) from animal, protist and fungal specimens were analyzed as described earlier, with an exclusion rate of 1%.

To cover several recently described phylum- and kingdom-level taxa and the genetic variation found within them, we downloaded raw (meta)genome and (meta)transcriptome data from several studies (21–25). All omics data were subjected to ITSx for correcting the orientation and recognizing the rRNA SSU, ITS and LSU marker regions. The 5′ SSU and 3′ LSU were recognized by cutadapt (26) using the corresponding probes 5′-CCTNGTTGATYCTGCCAGT-3′ and 5′-GCATTGTTGTTCCGATG-3′, respectively, allowing three mismatches. Chimera checking was performed separately for each marker using UCHIME. Chimeras with breaking points close to the SSU/ITS and ITS/LSU boundaries were recognized by comparing taxonomic assignments based on BLASTn results for each marker gene separately, using one of the earlier versions of the database as a reference. Taxa with conflicting matches at genus, order and kingdom levels—depending on taxonomic resolution—were removed as potential chimeras.

In addition to regular amplicons, we used target-captured rRNA marker amplicons from global soil and sediment samples (M. Hosseyni Moghaddam *et al*., unpublished results). These amplicons were processed, chimera-checked and indel-filtered as described earlier. We included only those target-capture amplicons that covered at least 800 bases of SSU, full-length ITS and/or at least 800 bases of LSU and met the aforementioned quality criteria. The newly included target capture, long-read metabarcoding and omics data were taxonomically identified based on BLASTn searches against EUKARYOME v1.6 using trimmed SSU, ITS and LSU sequences in separate queries. For these particular analyses, we trimmed SSU and LSU to 700 bases, discarding the 100 bases closest to the ITS region and further away than 800 bases from the ITS region, to fully match to the largest proportion of reference sequences. These analyses were repeated for the full-length SSU and LSU, and these assignments were used when they were found to be more informative than the trimmed counterpart, e.g. when the trimmed target region contained an intron.

For all types of input data, the full-length sequences were aligned with well-identified reference sequences as described earlier, followed by manual alignment correction and removal of positions with >90% gaps in ClipKIT v1.4.0 (27). Evolutionary model selection and maximum likelihood phylogenetic analysis with 1000 ultra-rapid bootstrap estimates were performed in IQ-TREE v2.2.2.6 (28). These phylogenies for SSU and LSU were used to spot any remaining chimeric sequences and to identify divergent taxa and erroneously identified taxa. Our taxonomic specialists were instructed to follow the same protocols (Supplementary Text S1).

To build a sizable database from the initial >10 million reads, we removed redundant, incomplete and low-quality reads. First, all reads (rRNA markers separately) were clustered at 100% sequence similarity. Identical reads were removed so that one read was retained per species. The ITS reads containing only ITS1 or ITS2, as recognized by ITSx, were removed. To build a smaller core dataset for chimera filtering of long-read data, only one longest read was retained per species and marker gene and ITS-only PacBio eDNA reads with >95% sequence similarity to the retained sequences were removed.

For the taxonomy of eukaryotes, we follow certain modifications to the original taxonomy implemented in INSDc (29). For metazoans and fungi, we use the versions modified in BOLD v4 (30) and Outline of Fungi (31), respectively. For protists, we use the taxonomy updates of PR2, except at kingdom level where we use a modification of Tedersoo (32) and Burki *et al*. (33). EUKARYOME makes consistent use of the main Linnaean ranks species—genus, family, order, class, phylum and kingdom—unlike SILVA and INSDc where the number of taxonomic ranks ranges from 1 to >20 depending on the rigor of systematic research on a particular taxonomic group. Additional ranks at the levels of subdomain, subkingdom and subphylum are maintained in the taxonomy database but not used in reference datasets. We hope that the use of comparable ranks will greatly simplify taxonomic understanding for users not accustomed to the intricacies of classification traditions across eukaryotic lineages—and that it will facilitate the use of traits databases that are linked to a text string related to species or genera in certain data fields. In EUKARYOME, the database and its underlying taxonomy are divided into zoological and botanical parts because of the biosystematics heritage. Although we are not in favor of this archaic split, the hundreds of identical genus names across the botanical and zoological worlds must be kept reliably separated. Such hemihomonyms misassigned to the plant or animal world are particularly common in taxonomic hierarchies of the SILVA database.

EUKARYOME also uses non-Latin coding for phylum, class and order-level monophyletic lineages of stramenopiles (34) and fungi (35) that have been rooted in the scientific literature and accepted as informal taxa. With a few exceptions, these taxa do not have taxonomically described and sequenced representatives. For the few taxa recently described in these groups, we use the formal taxonomy.

Taxonomic updates to the original INSDc data were retrieved from all three databases (SILVA, UNITE and PR2) and multiple additional sources. The updates in SILVA were preferred for plants and metazoans, updates for fungi were primarily sourced from UNITE, and updates for protists were primarily sourced from AlgaeBase (36) and PR2. For *Glomeromycota*, the updates in the specific and relatively smaller datasets of Outline of Fungi: *Glomeromycota* (31) and AMF-LSU (37) were prioritized over taxonomic assignments in other databases. Taxonomic identification of metabarcoding data in EUKARYOME has been continuously and iteratively updated in the course of eukaryote biodiversity studies (13, 14), where conflicting identifications are detected and updated or sequences are marked as chimeric and removed. The taxonomic updates are related to the version of the database and marker(s) used, making the versioning system of EUKARYOME an important component of its communication.

The EUKARYOME database is maintained by the Mycology and Microbiology Center, University of Tartu. New versions including additional data and taxonomic updates are planned every year. This is secured by our consortium of taxonomic specialists that actively work on metabarcoding data of various eukaryotic organisms. EUKARYOME welcomes new taxonomic specialist members, but it is not open for primary sequence data deposition. New data are added by database curators primarily from INSDc, UNITE, Joint Genome Institute (JGI) and specific metabarcoding and metagenomics studies producing long-read data, after careful quality control and taxonomic annotations.

## Results and discussion

**Figure 1.**
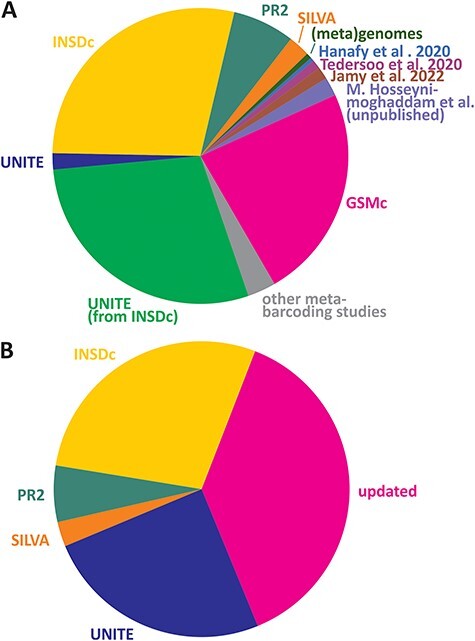
Relative contribution of read sources (**A**) and taxonomic assignment sources (**B**) in the EUKARYOME dataset. GSMc, Global Soil Mycobiome consortium dataset (41; V. Mikryukov *et al*., unpublished results).

Here, we introduce the EUKARYOME database that includes sequence data originating from several reference sequence databases and uniquely takes advantage of long reads for taxonomic placement of references derived from eDNA (Figure 1A). Genus-level information in the ITS marker improves identification for both the SSU and LSU, especially in cases where the rRNA genes have a low taxonomic resolution or when particular taxa are not sequenced for these genes (e.g. many groups of fungi). In addition, we use reliable higher-level phylogenetic placement of rRNA genes to resolve order- to kingdom-level uncertainties in the ITS data subset (13, 14). The latest taxonomic annotations in EUKARYOME are derived from multiple sources, relying on both original descriptions and multiple rounds of re-annotation and confirmation (Figure 1B).

**Figure 2.**
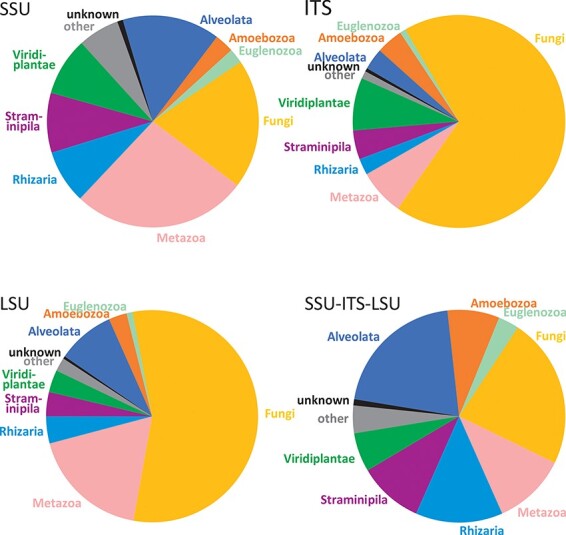
Comparison of kingdom-level taxonomic composition in data subsets of EUKARYOME: SSU, ITS, LSU and long-read SSU-ITS-LSU.

The dataset of EUKARYOME v1.8 contains a total of 1 294 684 entries covering all terrestrial and aquatic eukaryotic groups. These include 7831 duplicate entries from which putative introns of >29 bases are manually removed (accessions from which introns were removed are marked by a preceding underscore, e.g. _AY831412), as well as 2902 reads of rRNA genes from prokaryotes, mitochondria, plastids and nucleomorphs as outgroups. The EUKARYOME reference database covers nuclear rRNA SSU (178 967 sequences), ITS (1 059 787) and LSU (115 640) markers taken separately and combined (32 810 SSU-ITS-LSU reads). The differences in read numbers among markers roughly reflect molecular taxonomic and metabarcoding efforts using Sanger and PacBio sequencing in different taxonomic groups. Metazoans and protists are relatively well represented in the SSU dataset, although the fungal coverage is less rich. Fungi are, on the other hand, well represented in the ITS data subset, as are plants and certain protist groups such as oomycetes, rhodophytes and dinoflagellates. The LSU is more sporadically covered for certain fungal, metazoans and protist phyla, but these biases have been reduced by including eDNA long-read metabarcoding data (Figure 2). During the curation procedure, 4256 INSDc reads (0.4% of tested reads) are marked as being of low quality or chimeric (Supplementary Table S1).

**Figure 3.**
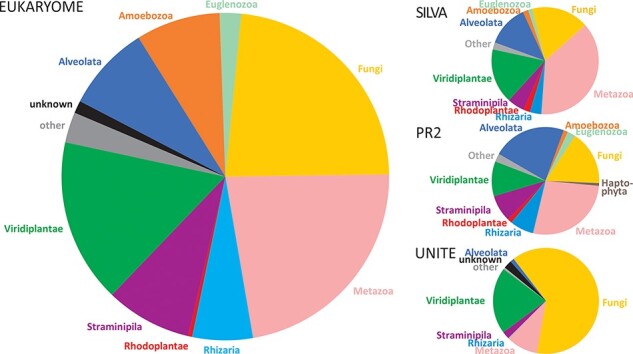
Comparison of kingdom-level taxonomic composition in the EUKARYOME core database and three other major reference sequence databases, SILVA v138.1 (46 577 SSU reads and 16 152 LSU reads), PR2 v4.14.1 (162 563 SSU reads) and UNITE v9.0 (Sanger sequence data subset; 2 367 811 ITS reads).

**Figure 4.**
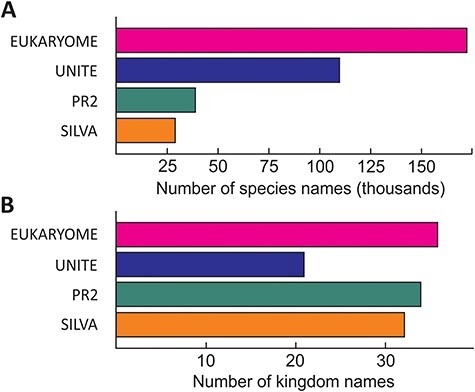
Relative richness of eukaryote species names (**A**) and kingdom names (**B**) in EUKARYOME and three other major reference sequence databases, SILVA (SSU and LSU), PR2 (SSU) and UNITE (ITS). Note that besides species names, UNITE harbors 793 114 species hypotheses.

The numbers of reads and their taxonomic representation in EUKARYOME are linked to the corresponding numbers in other nucleotide sequence databases, depending on how these databases handle redundant sequences and eDNA (Figure 3). EUKARYOME surpasses other databases in terms of taxonomic coverage (172 426 species names from 36 kingdom-level groups; Figure 4). This is explained by the broader taxonomic scope, the inclusion of three marker genes and the incorporation of recent data from (meta)genomics, (meta)transcriptomics and long-read metabarcoding studies.

**Figure 5.**
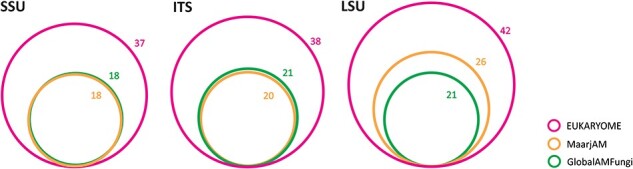
Relative richness and overlap of valid *Glomeromycota* genus names based on three marker genes in EUKARYOME, GlobalAMFungi and MaarjAM.

As an example, we provide an overview of an analysis of the subset of the fungal phylum *Glomeromycota*, which is the largest group of arbuscular mycorrhizal fungi and sustains mineral nutrition of most terrestrial plant species. Because of its ecological importance, thousands of studies have specifically addressed this group. While molecular taxonomic and barcoding efforts mostly focus on the ITS and LSU, roughly half of the metabarcoding studies utilize the short V4–V5 subregion in the SSU. To communicate the nameless molecular taxa, a ‘virtual taxon’ (VT) system that nominates type sequences has been established in the MaarjAM (38) and GlobalAMFungi (39) databases. Using long reads, EUKARYOME links the three marker genes and provides Latin taxon names to SSU reads using a phylogenetic approach. The 18 valid genera in MaarjAM and GlobalAMFungi are increased to 37 for the SSU marker in EUKARYOME. However, the ITS and LSU markers contain much more named genera—38 and 42, respectively (Figure 5)—and twice as many named species. Based on manual quality-filtering, 21.6% of SSU reads, 17.2% of ITS reads and 16.9% of LSU reads in INSDc and MaarjAM are marked as of low quality or chimeric. In particular, 15.1% of VT type sequences are considered to be of substandard quality or otherwise compromised. Roughly half of the VTs are scattered within one genus or two closely related genera based on a Maximum Likelihood phylogeny of the SSU, whereas another half of putative phylotypes are unrepresented by VTs suggesting undocumented taxa (Supplementary Figure S1). The relatively large quality issues in *Glomeromycota* SSU may be related to its poor taxonomic resolution and cloning prior to Sanger sequencing. From the reference database, taxonomic information and taxonomic resolution perspectives, we recommend the use of ITS and/or LSU for *Glomeromycota* metabarcoding and splitting the SSU-based VTs into species-level groups based on long reads.

EUKARYOME is maintained in a spreadsheet format for rapid searches and the possibility of updating individual data fields using routine options of MS Excel and other spreadsheet programs or text-editing programming languages. It includes the following data fields (columns separated by semicolon): nomenclature (zoological or botanical), origin of the data, origin of the latest taxonomic identification or confirmation (including versioned updates by the EUKARYOME curators), read coverage (SSU, ITS, LSU, SSU-ITS, ITS-LSU or all), the full-length read and taxonomy at the levels of kingdom, phylum, class, order, family, genus and the species epithet (including any subspecies-level classification separated by dots). Taxonomic fields below the level of identification are marked as dots for maintaining the database structure when converting among different formats. For preparing the BLASTn database for various pipeline-specific formats, EUKARYOME is converted to the fasta format, with data fields separated by colons. The nomenclature and sequence origin fields are excluded from the fasta-formatted files.

The reference datasets of EUKARYOME can be accessed and downloaded from the homepage, http://www.eukaryome.org. Users can select multiple dataset types and formats according to their needs. Dataset types include data subsets for each rRNA marker and the long read covering all these markers. Based on the main fasta-formatted version, users can easily exclude unwanted data and build their own data subsets for custom analysis. All data fractions are formatted into QIIME2, mothur, DADA2 and UCHIME, which are the most commonly used analytical pipelines or stand-alone programs.

EUKARYOME issues its own accession numbers (‘EUK’ followed by seven digits, e.g. EUK1210054), which are limited to reads originating from metabarcoding, (meta)genomics and (meta)transcriptomics datasets and lack previous accession numbers in INSDc, UNITE or JGI. These reads along with the basic metadata (isolation source, country and geographical coordinates as available) can be accessed from the EUKARYOME homepage in a spreadsheet format and over the PlutoF platform (40). PlutoF offers third-party curation service to add source metadata, taxonomic annotations and evaluate read quality.

In conclusion, EUKARYOME serves as a comprehensive, curated reference sequence database of SSU and LSU genes and long rRNA markers for quality-filtering and taxonomic identification of metabarcoding and omics data. It is the first such database that systematically collects high-quality, third-generation sequencing data from PacBio and Oxford Nanopore (consensus reads) platforms. We anticipate that growth of the database and taxonomic updates will occur incrementally as new long-read sequence data accumulate and as INSD and other source databases undergo re-annotation. Also, single-cell genomics and transcriptomics of microeukaryotes will probably offer high-quality reference sequence data for multiple divergent, uncultured lineages (25). In addition to ongoing improvements in the accuracy of taxonomic identification of eukaryotes and adding long-read data, future developments will focus on additional markers such as the rRNA intergenic spacer region, mitochondrial genes and functional genes as well as offering well-annotated, long-read reference alignments for phylogenetic placement algorithms.

## Supplementary Material

baae043_Supp

## Data Availability

The sequences are deposited in public sequence databases, INSDc and UNITE. All sequences, including the long reads obtained from other sources, are available in spreadsheet format over the EUKARYOME homepage (corresponding to FAIR - findability, accessibility, interoperability, and reusability - data requirements). Sequences introduced to EUKARYOME from eDNA are equipped with principal metadata about the source of isolation and geographical locality if known. Versions of the EUKARYOME database and related explanatory files can be downloaded from http://www.eukaryome.org. Multiple sequence alignments of kingdom- and phylum-level groups, used for preparing ML trees, are available in EUKARYOME homepage.
