# Supplementary material for: EUKARYOME: the rRNA gene reference database for identification of all eukaryotes
Source: Database (Oxford). 2024 Jun 12;2024:baae043. doi: 10.1093/database/baae043 (PMC11168333; doi:10.1093/database/baae043)
Supplement: baae043_Supp [file baae043_supp.zip › suppl_data/Graphical abstract.pdf]

EUKARYOME the rRNA gene reference database for identification of eukaryotes

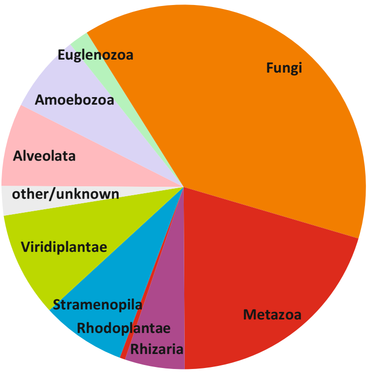

EUKARYOME

- 18S rRNA gene
- 28S rRNA gene
- rRNA ITS region
- rRNA long-read

http://www.eukaryome.org
