# Supplementary material for: EUKARYOME: the rRNA gene reference database for identification of all eukaryotes
Source: Database (Oxford). 2024 Jun 12;2024:baae043. doi: 10.1093/database/baae043 (PMC11168333; doi:10.1093/database/baae043)
Supplement: baae043_Supp [file baae043_supp.zip › suppl_data/Text S1 EUKARYOME instructions.pdf]

Text S1. Instructions to EUKARYOME annotators and curators for quality and taxonomic assignments.

List of tasks:

1. Intron removal of SSU and LSU reads for maintaining a copy of intron-free reads in EUKARYOME
2. Building of phylum- and/or kingdom level phylogenetic trees
3. Taxonomic annotation of reads: checking previous annotations and fixing issues

Required programs and their sources:

1. MAFFT (for preparing secondary alignments). Use online source with default options: https://mafft.cbrc.jp/alignment/server/
2. ALIview (for viewing and editing alignments. It outperforms other programs such as SeAl, SeaView and MacClade). Download and install the latest version from https://github.com/AliView/AliView
3. ClipKIT (for trimming final alignments). Use the online version (works if the aligned fasta file is <10 Mb) or, better, download and install into your computer. https://github.com/JLSteenwyk/ClipKIT (note: linux version is easier to handle than Windows version)
4. IQ-tree (for tree building; outperforms RaXML and PhyML). For the size of our trees, please download and install into your computer. http://www.iqtree.org/ (note: linux version is easier to handle than Windows version)
5. FigTree (for viewing output trees; other alternative software can be considered). http://www.softsea.com/review/FigTree.html

Input files sent to you:

1. Spreadsheet file including the accessions, preliminary identifications and original, untrimmed sequences
2. Primary fasta-formatted alignment file (using the data in the spreadsheet, aligned using MAFFT, and checked for problematic reads)

Recommended workflow for non-bioinformatician:

1. Open the alignment file using AliView. You will see that it also includes some “outgroup” sequences, which should be kept for phylogenies. Try to locate the start and end of SSU, 5.8S, and start of LSU. See the figures below. Note that (meta)genomic sequences have long accessions and PacBio metabarcoding accession in EUKARYOME start with “EUK…….”

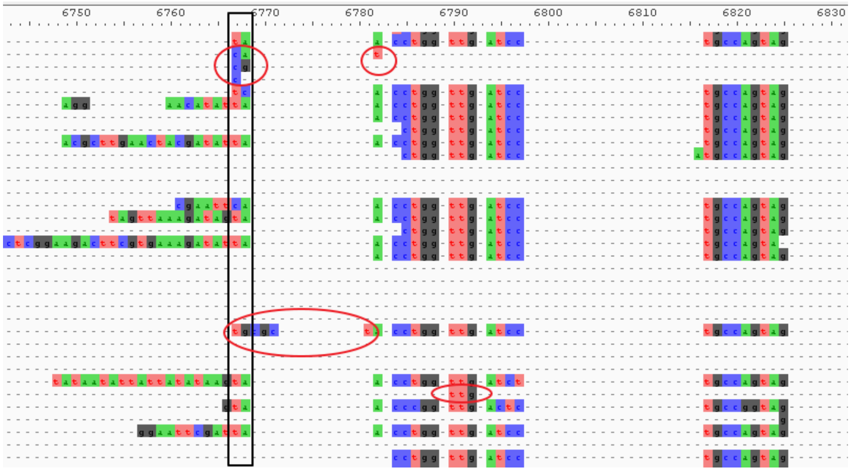

Figure. Start of SSU. The box indicates start of the SSU at “TA” (rarely other) nucleotides (note these are the first well-aligned cols). Red ellipses indicate alignment errors by MAFFT. Here, please remove all nucleotides left from the column.

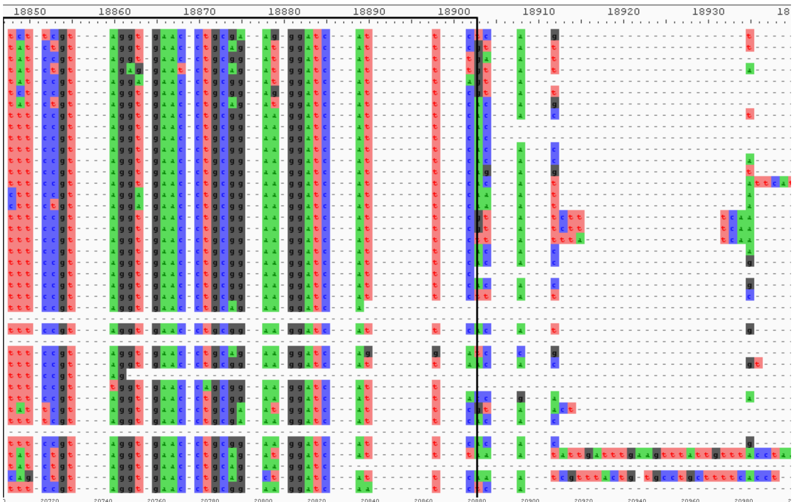

Figure. End of SSU (and start of ITS1). SSU (column-bordered) ends with a motif of "GGAAGGATCATTA" or similar (note that these are usually the last well-aligned cols before ITS1).

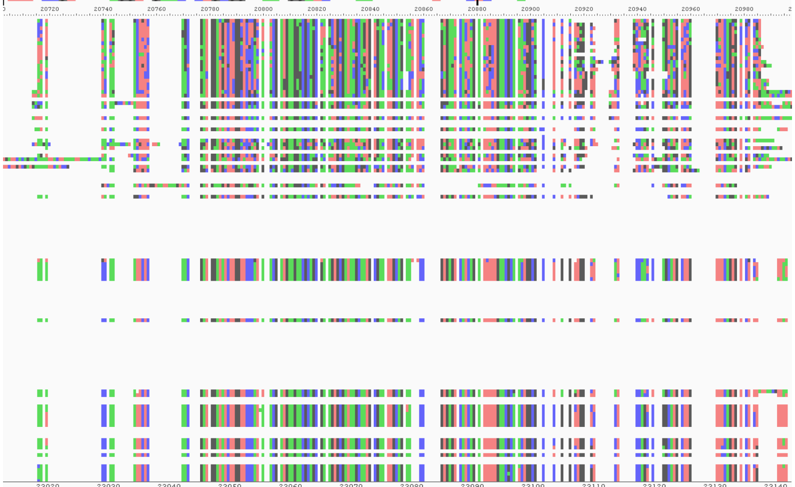

Figure. The 5.8S rRNA gene is a small alignable island within the ITS region. The immediate start and end are difficult to recognize.

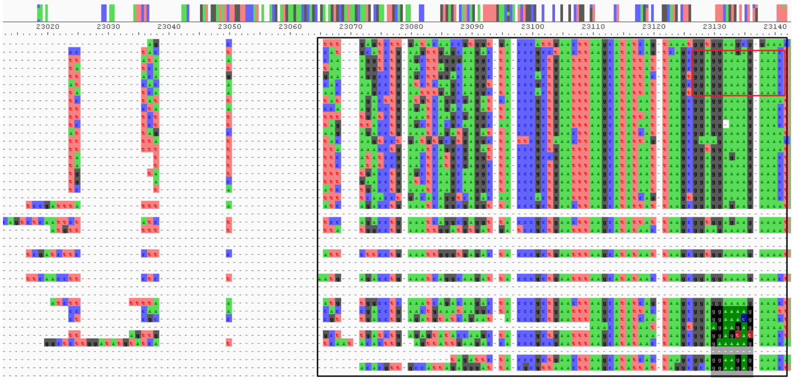

Figure. The start of LSU (delimited by column; roughly the first alignable area after the ITS region. The highly conserved motif "GGAGGAAAAGAAA" (in red box) some 40 bp from the ITS can be visually easily recognized.

A. Save your file in every ca 15 minutes. If the computer crashes or you mistakenly delete unintended reads, you can get back to the recent situation
B. I already deleted the sequences left in SSU and right in LSU (i.e. outside the region of interest) and most end-introns. Scroll right to the alignment and find internal introns. Here, let's define an intron as any unique stretch of a sequence >29 bp in length (sometimes thousands of bases long; see below). In this position, >90% of other accessions have gaps only. Most introns start from a random position, but there are positions where introns are very common (these are quite long introns). Introns typically have long homopolymers, dinucleotide repeats or they are very rich in A/T nucleotides. When you reach the ITS region (including the 5.8S gene), do not trim further as there are (almost) no introns. Proceed with introns in the LSU region
In the end of LSU, it is very difficult to recognize introns and where the coding sequence ends. So, it is recommended that you finish intron removal when you reach a point where most "EUK"-encoded sequences end (there is the reverse primer site for reads from Jamy et al. 2022).

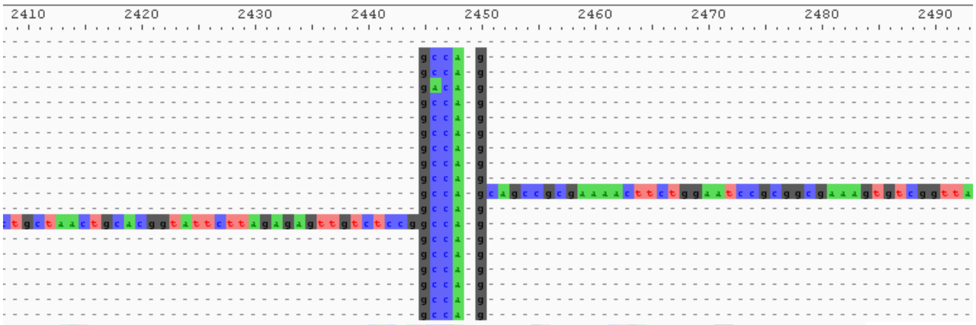

Figure. Typical introns starting off from conserved positions. These introns need to be deleted.

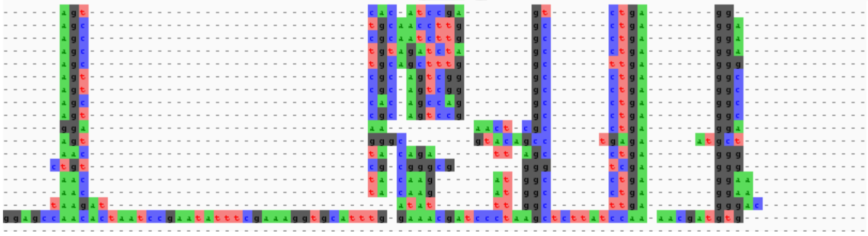

Figure. A nasty intron in the bottom (i.e. aligned badly). Please do not try to remove such introns as we do not understand where it actually starts and ends.

2. Open the alignment file in Notepad or equivalent (not MS Word!), and copy the edited alignment to MAFFT window. Using default options, please run the second round of aligning. Get back to the secondary alignment in AliView and remove remaining introns (the nasty ones often turn to "regular ones": usually 5-10% have remained "hidden" in the first round (this is because several introns of different origin may coincide and when removing the main one, other will be exposed in the second run). Please save the file adding "intronsremoved" in the end.
3. Continue with the same alignment for tree building purpose but rename it differently immediately. It is recommended that you remove the regions that the program could not align with confidence. These include some of the variable (V) regions of SSU and LSU and the ITS1 and ITS2 spacers. You are recommended to retain 5.8S as it keeps extra phylogenetic information.
   From individual reads, please also remove the end regions IF these are not properly aligned to the rest of the sequences. There may be as much as 10% of reads that end with a partial intron. Including these may greatly weaken your phylogeny. See below.

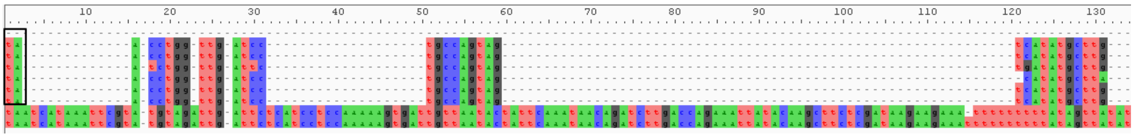

Figure. The start of SSU (box) with an intron shared by two sequences from the same samples. The intron should be deleted as far right when it reliable aligns to the rest of sequences.

4. When you are happy with the overall alignment, please run it through ClipKIT to remove positions with too little information. For this purpose, ClipKit "gappy" works best, with 0.9 (90% losses allowed) default option providing reasonable results.

   Command line: clipkit -m gappy <inputfile.fas>

   If interested, you can play with the gappiness parameter between 0.8 and 0.99 and see some changes in tree performance.
5. Run the trimmed alignment using IQ-tree:

```
Command line: iqtree -s Fungi1clipped.fas -m TEST -bb 1000 -alrt 1000 -T
AUTO
```

Here -bb and -alrt indicate inclusion of different types of bootstrap support, which is necessary for decision making! Use minimum of 1000/1000 bootsrap reps (IQ-tree allows no less). -m indicates testing for best suitable evolutionary model; -T AUTO defines “optimal selection” of cores for calculations.
It usually takes 1-2 days to complete. Interrupted jobs can be revived.

6. Visualise the alignment using FigTree (or equivalent).
   A. When opening, define the extra values as bootstrap support
   B. Re-root the alignment at the relevant outgroup. You may see that some taxa considered as ingroup fall outside this. These are probably misidentified at the level of kingdom/phylum. Also be vary of the long-branch-attraction issue!
   C. order the tree “to decreasing”. On the left, select “Trees” -> click “order nodes” -> select “decreasing”
   D. View bootstrap support values on branches. On the left, tick “branch labels” -> select “display” and “bootstrap support”
   E. Zoom in slightly (top-left button) to get a rough overview of the tree
   F. Take a detailed view of the tree by starting from the top and colouring / highlighting branches of interest (for this, switch from “Node view” to “Clade view” by pressing the top-central button. Make notes on every misidentified or insufficiently identified taxon into the spreadsheet file. This can be done easiest by typing the accession to search in the Excel-opened spreadsheet. To column C, please add the new taxon name. To column D, please add the rank of this taxon name (k=kingdom, p=phylum, c=class, o=order, f=family, g=genus; s=species). For assigning a species or genus name, you must be VERY confident about this (i.e., here your expertise is irreplaceable). If you are satisfied with the taxonomic information present (hopefully in most cases), add nothing.

	A	B	C	D	E
1	Fasta_taxonomy_string	Original_sequence	Taxonomic_update	Update_rank	Quality_comments
2	>KC670142;cfFungi;	TACCTGGTTGATTCT(	Rozellomycota	p	lowquality
3	>GU001154;Mantamonada;Mantamonadia;Mai	CATATGCTTGTCTCA.	Mantamonada	k	chimericpossibly
4	>EUK1202143;Eukaryota_kgd_incertae_sedis;	CACATATCAGAGTG(	Tetramitia	o	longbranch
5	>GU290077;cfCryptista;	ACCTGGTTGATCCTG	Rhodoplantae	k	
6	>EUK1211968;Eukaryota_kgd_incertae_sedis;	ATGGCGATCACGGG	Fungi;Group2	p	

Figure. Example of the input table (highlighted) fields and annotations (cols C-E). In C6: an example, how novel phylum or class-level clades could be named if you wish to group the undescribed taxa.

   G. In column E, you may have information about the read quality or suspected chimerism. Chimeric reads typically have a very long unique branch compared with the closest relative (parent read). If you do not feel qualified, do not bother.
   H. OPTIONAL. When going through the tree, you have probably noticed an unexpected number of taxa that do not fall into any phylum or class. This is complicated, because these may represent truly undiscovered taxa but also very bad quality sequence, putative pseudogene, bad alignment (e.g. introns forced to align to conserved region) or an “outgroup” sequence sneaking in, or even a regular sequence connected through a chimera. If you feel confident, You may provide tentative numbers for the unrecognized clades, especially if these are comprised of >1 sequence.
7. Please return to me the following files:
   A. Filled spreadsheet file (added information about taxonomic changes and perhaps quality; based on this, I will take the changes into the database)
   B. Tree file (newick formatted)
   C. Alignment file where ONLY introns have been removed (for making an intron-free copy also available in the database)
   D. Final alignment file used for tree building
   We would normally upload the final alignment file and tree files to the EUKARYOME homepage.
