# Supplementary material for: EUKARYOME: the rRNA gene reference database for identification of all eukaryotes
Source: Database (Oxford). 2024 Jun 12;2024:baae043. doi: 10.1093/database/baae043 (PMC11168333; doi:10.1093/database/baae043)
Supplement: baae043_Supp [file Figure S1 Glo9SSU_figtree.pdf › Figure S1 Glo9SSU_figtree.pdf]

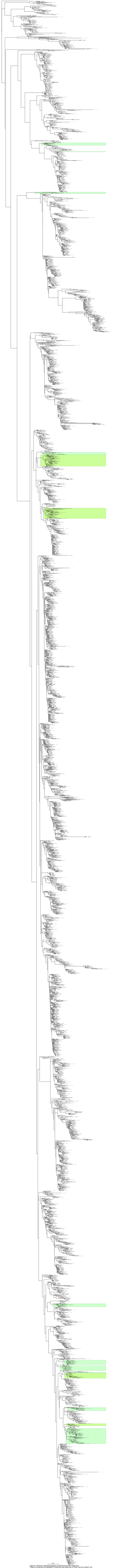


Figure S1. Maximum Likelihood phylogram of Glomeromycota SSU sequences.
Putative novel genera are highlighted. Problematic type sequences of Virtual Taxa are marked in red.
